# The no-go zone: a qualitative study of access to sexual and reproductive health services for sexual and gender minority adolescents in Southern Africa

**DOI:** 10.1186/s12978-018-0462-2

**Published:** 2018-01-25

**Authors:** Alex Müller, Sarah Spencer, Talia Meer, Kristen Daskilewicz

**Affiliations:** Gender, Health and Justice Research Unit, University of Cape Town, Observatory, Cape Town, South Africa

**Keywords:** Adolescents, Sexual and reproductive health and rights, Sexual and gender minorities, HIV, Service provision, Heteronormativity, Criminalisation, Stigma, Southern Africa, LGBT

## Abstract

**Background:**

Adolescents have significant sexual and reproductive health needs. However, complex legal frameworks, and social attitudes about adolescent sexuality, including the values of healthcare providers, govern adolescent access to sexual and reproductive health services. These laws and social attitudes are often antipathetic to sexual and gender minorities. Existing literature assumes that adolescents identify as heterosexual, and exclusively engage in (heteronormative) sexual activity with partners of the opposite sex/gender, so little is known about if and how the needs of sexual and gender minority adolescents are met.

**Methods:**

In this article, we have analysed data from fifty in-depth qualitative interviews with representatives of organisations working with adolescents, sexual and gender minorities, and/or sexual and reproductive health and rights in Malawi, Mozambique, Namibia, Zambia and Zimbabwe.

**Results:**

Sexual and gender minority adolescents in these countries experience double-marginalisation in pursuit of sexual and reproductive health services: as adolescents, they experience barriers to accessing LGBT organisations, who fear being painted as “homosexuality recruiters,” whilst they are simultaneously excluded from heteronormative adolescent sexual and reproductive health services. Such barriers to services are equally attributable to the real and perceived criminalisation of consensual sexual behaviours between partners of the same sex/gender, regardless of their age.

**Discussion/ conclusion:**

The combination of laws which criminalise consensual same sex/gender activity and the social stigma towards sexual and gender minorities work to negate legal sexual and reproductive health services that may be provided. This is further compounded by age-related stigma regarding sexual activity amongst adolescents, effectively leaving sexual and gender minority adolescents without access to necessary information about their sexuality and sexual and reproductive health, and sexual and reproductive health services.

## Abstrait

**Contexte:**

Les adolescents ont des besoins importants en matière de santé sexuelle et reproductive. Cependant, des cadres juridiques complexes et des attitudes sociales concernant la sexualité des adolescents, y compris les valeurs des prestataires de soins, régissent l’accès des adolescents à ces services. Ces lois et attitudes sociales sont souvent antipathiques aux minorités sexuelles et de genre. La littérature existante présuppose que les adolescents s’identifient comme hétérosexuels et se livrent exclusivement à des activités sexuelles (hétéronormatives) avec des partenaires du sexe/ genre opposé. Nous ne savons que très peu si et comment les besoins des adolescents appartenant à une minorité sexuelle ou de genre sont satisfaits.

**Méthodes:**

Dans cet article, nous avons analysé cinquante entretiens qualitatifs approfondis avec des représentants d’organisations travaillant avec des adolescents, des minorités sexuelles et de genre et / ou des droits et santé sexuels et reproductifs au Malawi, au Mozambique, en Namibie, en Zambie et au Zimbabwe.

**Résultats:**

Dans ces pays, les adolescents appartenant à une minorité sexuelle et/ ou de genre connaissent une double marginalisation en matière d’accès aux services de santé sexuelle et reproductive: en tant qu’adolescents, ils rencontrent des obstacles pour accéder aux organisations LGBT, qui craignent d’être qualifiés de «recruteurs d’homosexualité»; alors qu’ils n’ont déjà pas accès aux services hétéronormatifs de santé sexuelle et reproductive pour adolescents. De tels obstacles aux services sont également attribuables à la criminalisation réelle et perçue des comportements sexuels consensuels entre partenaires de même sexe / genre, quel que soit leur âge.

**Discussion / conclusion:**

La combinaison de lois criminalisant l’activité consensuelle de même sexe / genre et la stigmatisation sociale envers les minorités sexuelles et de genre fait que les services légaux de santé sexuelle et reproductive pouvant être fournis ne sont pas accessibles. Cette situation est. encore aggravée par la stigmatisation liée à l’âge chez les adolescents, laissant ainsi les adolescents des minorités sexuelles et de genre sans accès aux informations nécessaires sur leur sexualité et leur santé sexuelle et reproductive, ainsi que sur les services de santé sexuelle et reproductive.

## Abstrato

**Contexto:**

Os adolescentes têm altas necessidades de saúde sexual e reprodutiva. No entanto, quadros jurídicos complexos e atitudes sociais sobre a sexualidade dos adolescentes, incluindo os valores dos cuidadores, governam o acesso dos adolescentes a esses serviços. Essas leis e atitudes sociais são muitas vezes hostis às minorias sexuais e de gênero. A literatura existente pressupõe que os adolescentes se identificam como heterossexuais e se envolvem exclusivamente na atividade sexual (heteronormativa) com parceiros do sexo / gênero opostos. Sabemos muito pouco sobre se e como as necessidades dos adolescentes pertencentes a uma minoria sexual ou de gênero estão satisfeitas.

**Métodos:**

Neste artigo, analisamos cinquenta entrevistas qualitativas em profundidade com representantes de organizações que trabalham com adolescentes, minorias sexuais e de gênero e / ou direitos e saúde sexual e reprodutiva no Malawi, Moçambique, Namíbia, Zâmbia e Zimbábue.

**Resultados:**

Nesses países, os adolescentes pertencentes a uma minoridade sexual e / ou de gênero experimentam uma dupla marginalização no acesso a serviços de saúde sexual e reprodutiva: como adolescentes, enfrentam obstáculos ao acesso a organizações LGBT que temem ser chamados de “recrutadores homossexuais”; enquanto eles ainda não têm acesso a serviços de saúde sexual e reprodutiva heteronormativos para adolescentes. Tais barreiras aos serviços também são atribuíveis à criminalização real e percebida do comportamento sexual consensual entre parceiros do mesmo sexo/ gênero, independentemente da idade.

**Discussão / Conclusão:**

A combinação de leis que criminalizam a atividade consensual do mesmo sexo / gênero e estigma social em relação a minorias sexuais e de gênero significa que os serviços para saúde sexual e reprodutiva que podem ser fornecidos legais não são acessíveis. Esta situação é ainda agravada pelo estigma relacionado com a idade entre os adolescentes, deixando os adolescentes de minorias sexuais e de gênero sem acesso à informação necessária sobre sua sexualidade e sua saúde sexual e reprodutiva, bem como sobre os serviços de saúde sexual e reprodutivo.

## Plain English summary

Little is known about how adolescents who are sexual or gender minorities (who may identify on the LGBT spectrum) access services for sexual and reproductive healthcare in Southern African countries This is because existing literature either assumes all adolescents are straight and cisgender or does not specifically ask about the sexual and gender identities of adolescents in their studies.

In order to find out about access to sexual and reproductive health services for sexual and gender minority adolescents, we conducted fifty interviews with people who provide sexual and reproductive health services, or who provide services to sexual and gender minority people (LGBT organisations) in Malawi, Mozambique, Namibia, Zambia and Zimbabwe.

We found that adolescents struggle to access services at LGBT organisations. This is because LGBT organisations mainly work with adults, because they fear being seen as promoting alternative sexuality to young people. Adolescents also struggle to access services at other organisations that specialise in sexual and reproductive health services, because these organisations do not cater to sexual and gender minorities, and may even be trans- or homophobic.

The combination of laws that criminalise consensual same sex/gender activity, and the social stigma towards sexual and gender minorities makes providing services to sexual and gender minority adolescents even more difficult. This is worsened by taboos against teenage sexuality as bad or immoral, and means that sexual and gender minority adolescents have little to no access to necessary information about their sexuality and sexual and reproductive health, and sexual and reproductive health services.

## Background

Adolescents have significant needs related to HIV and sexual and reproductive health (SRH). Across Southern Africa, the birth rate amongst girls aged 15 to 19 years ranges from 8.2% in Malawi to 16.7% in Mozambique [1, 2]. Similarly, HIV/AIDS is now recognised as the primary reproductive health concern for teenagers, with young women (15 to 24 years of age) accounting for 26% of all new infections in the Eastern and Southern African region in 2016 [3, 4]. Given that adolescence is typically the period of sexual debut, good SRH services can significantly contribute to the prevention of unplanned pregnancy and HIV, as well as to limiting exposure to sexual violence and coercion [5]. Furthermore, with a generation of peri-natally HIV-infected adolescents now reaching sexual maturity due to increasing access to antiretroviral therapy, the integration of HIV and SRH services becomes even more important. Adolescents living with HIV have health needs particular to their age demographic and context, and behavioural, medical, and social interventions and services must therefore be varied and specialised [3].

Legislative frameworks regulate adolescent sexuality and the provision of SRH services to adolescents. A number of ‘ages’ are legally determined: the most important in the context of SRH are the age at which adolescents can seek and consent to medical services without parental consent and the age at which adolescents are legally allowed to have sex. In many countries in Southern Africa, these ages are not aligned, leading to contradictions in the legal framework when, for example, a 15-year old teenager is not legally allowed to have sex, but is allowed to access contraception and HIV testing [6]. Further, these ‘ages’ not only determine what adolescents (legally) can and cannot do, but they also determine protective duties for healthcare providers, such as mandatory reporting requirements in cases where providers have knowledge of adolescent underage sex. In addition to the complex legal provisions, healthcare providers’ own morals and values influence their service provision by determining when adolescents are ‘worthy’ or ‘old enough’ to access certain SRH services [7].

Most research evidence and policy responses on Southern African adolescents’ HIV and SRH share one crucial shortcoming: they assume that adolescents identify as heterosexual, and exclusively engage in (penetrative, penis-vagina) sexual activity with partners of the opposite sex/gender.^1^ Adolescents identifying as lesbian, gay or bisexual (sexual minority adolescents) and adolescents identifying as transgender or gender non-conforming (gender minority adolescents)^2^ are often not recognised in SRH research and interventions, nor are their specific needs addressed (see, for example [5, 8–10]). An illustrative example is a report reviewing adolescents’ sexual and reproductive health in Zimbabwe [11], which does not explicitly mention the sexual orientation or gender identities of the adolescents in question (and thus assumes them to be heterosexual and cisgender), nor whether health services address any non-normative SRH needs. This is common in programmes and research on adolescents. Articles that do mention sexual orientation simply list it as a factor that “marginalize[s] [adolescents] because of individual characteristics” but fail to outline or address substantial issues that pertain to the HIV and SRH needs of sexual and gender minority adolescents [12].

The underlying assumption of heterosexuality, and the subsequent omission of sexual and gender minority adolescents in research on adolescent SRH in Southern Africa, is indicative of wider societal heteronormativity [13], including in healthcare provision [14, 15] and research [16]. Heteronormativity is a social construct that assumes heterosexual identities and relationships are the norm, and thus makes sexual and gender minority identities invisible in all facets of social life, including in healthcare provision and research [13, 17]. Through its pervasive normative influence, it leads people, in and outside of the healthcare sector, to assume that every patient, healthcare provider, student, research participant, and researcher is heterosexual and cisgender. As a result, sexual and gender minority people’s diverse health needs are not routinely recognized [18], documented [19], taught [20] or researched [21].

Despite advances in sexual orientation-related anti-discrimination laws globally, many countries still criminalise consensual same-sex activity [22]. In many Southern African countries, these laws originate from Victorian penal codes, introduced during British colonial rule, and offences are defined as ‘sodomy’ or ‘carnal knowledge against the order of nature’ [23]. The enforcement of such laws is inconsistent [24] and dependent on national political and social contexts. Regardless of enforcement, however, laws that criminalise same-sex behaviour effectively marginalise sexual and gender minority individuals. Although evidence from African countries is scant [25], public health research from other contexts shows that criminalisation and minority status lead to poorer health outcomes ranging from infectious disease to mental health [26]. Ironically, health policies in some of the same countries that criminalise same-sex activity categorise ‘men who have sex with men’ as ‘key populations’, indicating their necessity for health services due to their increased vulnerability to HIV resulting from social exclusion and criminalising legal frameworks [27]. This vulnerability is confirmed by a study from South Africa (where there is no criminalisation) with both heterosexual and sexual minority teenagers: compared to heterosexual matched peers, sexual minority teenagers experienced higher levels of partner-perpetrated violence, and showed higher levels of depression, traumatic stress and substance use [28]. Whilst there is no current evidence, it is probable that these differences are even more pronounced in countries where same-sex activity is criminalised.

Given these restrictive legal frameworks around same-sex activity, as well as the invisibility of sexual orientation and gender identity in current adolescent sexual and reproductive health policies, our paper examines the current provision of SRH services, which include HIV-related services, to sexual and gender minority adolescents in five Southern African countries: Malawi, Mozambique, Namibia, Zambia, and Zimbabwe. Table 1 provides an overview of the current legal and policy framework related to same-sex activity in the five countries that our study was conducted. Included in this study is Mozambique, a country where consensual same-sex activity was decriminalised in 2014, as well as four countries with differing levels of criminalisation.

**Table 1:** Legal framework for sexual and gender minority adolescents in study countries

	Where in the law?	Male same sex activity criminalized	Female same sex activity criminalized	Enforced through arrests	‘Promotion’ or ‘morality’ law	Reporting duty for healthcare providers
Malawi	Penal Code Cap. 7:01, Sections 137A, 153, 154, 156	Yes “Against the order of nature”, “Attempted unnatural offence”, “Gross male indecency”	Yes “Gross female indecency”	Yes	No	No
Mozambique	No	No	No	No	No	No
Namibia	Roman Dutch common law (based on case law), no codified provision	Yes (not codified)	No	No	No	No
Zambia	The Penal Code Act (amended by Act 15 of 2005), Sections 155, 156, 15	Yes “Against the order of nature”, “Attempted unnatural offence”, “Gross indecency” Imprisonment of 7-14 years for adults, community service or counseling for children under the age of 18	Yes “Gross indecency” Imprisonment of 7-14 years for adults, community service or counseling for children under the age of 18	Yes	No	No
Zimbabwe	Criminal law (Codification and reform) Act Section 73	Yes “Sodomy” Fine and/ or imprisonment up to one year	No	Yes	No	No

For the purposes of this research, adolescents were considered those aged 12 to 18. We argue that sexual and gender minority adolescents in these countries experience double-marginalisation due to their age and sexual and/or gender minority identity, and that they are routinely excluded from existing SRH services.

## Methods

### Study design, sampling and recruitment

We conducted a qualitative interview-based study with representatives of organisations working with, or providing SRH services to adolescents in Malawi, Mozambique, Namibia, Zambia and Zimbabwe. These organisations do not have a specific focus on sexual and gender minority adolescents. In this paper, we refer to them as ‘(A)SRH organisations’ – to signify provision of either specific adolescent sexual and reproductive health (ASRH) services, or general SRH services that are also open to adolescents. Additionally, in each of the five countries, we interviewed representatives of organisations focused on advocacy for, or services to, sexual and gender minority people – widely referred to as LGBT^3^ (lesbian, gay, bisexual and transgender) organisations. Given that same-sex activity is criminalised in four out of the five countries in this research, all except Mozambique, and that, additionally, adolescent sexuality, homo- and bisexuality as well as gender non-conformity is heavily stigmatised in all five countries, we decided to interview these NGO representatives as proxies in order to protect sexual and gender minority adolescents from potential harms. Further, NGO representatives are in a better position to talk about pathways and barriers because they have the accumulated experiences of having worked across sectors at both service delivery and policy levels. Thus they are able to draw comparisons and better evaluate the impact of the law and policy framework on sexual and gender minority ASRH access. In order to gain better insight, where possible, we also interviewed government representatives involved in policymaking on issues of ASRH.

Potential participants were identified through three methods: (1) from a contact list of non-governmental organisations working with, or providing services to adolescents in each of the five countries that the researchers had compiled during the years of their work on SRH in Southern Africa; (2) by participants electing to be contacted for further research at the end of a related online survey of (A)SRH NGO workers, and (3) through referrals from the researchers’ networks and participants themselves. We purposively sampled participants based on these three methods by contacting potential participants by email or phone and asking them to participate in the research.

### Data collection

Qualitative interview data were collected from October 2015 to May 2016. In total, we conducted 50 interviews with 51 participants: seven participants in Malawi, nine in Mozambique, nine in Namibia (one interview had two participants), 13 in Zambia, and 13 in Zimbabwe (see Table 2 for more detail). Due to local proficiency and the pervasive use of English in the NGO sectors in Malawi, Namibia, Zambia, and Zimbabwe, researchers (AM, KD, TM and SS) interviewed participants in person in English. Two researchers were present for most interviews and all interviews were recorded and then transcribed. Additionally, the second researcher took notes during the interviews. One interview was conducted over the phone with a participant based in Malawi.

**Table 2:** Affiliation of participants, by country

Country	Adolescent/ SRH organisation	LGBT organisation	Policy maker	Total Participants
Malawi	4	2	1	7
Mozambique	8	1	–	9
Namibia	6	2	1	9
Zambia	11	1	1	13
Zimbabwe	10	1	2	13

All interviews in Mozambique were conducted by an experienced research consultant through phone interviews in Portuguese which were recorded, transcribed and then translated into English.

### Data analysis

All transcripts were imported to NVivo 11 for coding. A team of three researchers (AM, KD, SS) developed a coding framework based on the research questions, interview guide, and interview notes. Each researcher independently coded three transcripts using the coding framework. Afterward, the team discussed the coding process to identify any discrepancies in coding between the researchers and refined the codes. Thereafter, one researcher (AM) was responsible for coding the 50 transcripts for sexual orientation and gender identity-related codes. All coded data were read for similarities and the emergence of key themes, which then guided a further thematic analysis.

### Research ethics

The University of Cape Town’s Faculty of Health Sciences Human Research Ethics Committee approved this study (HREC reference number 683/2015). All participants understood that participation was voluntary, completed an informed consent process and signed an informed consent form prior to participating. For this paper, we use pseudonyms, and all responses have been anonymized.

## Results

### Attitudes towards sexual and gender minority adolescents

Participants from (A)SRH organisations were heterogeneous in their attitudes towards sexual and gender minority adolescents. Some affirmed sexual diversity and recognised that sexual and gender minority adolescents should receive SRH services without judgement. For example, Rutendo describes engaging with her colleagues during a training about providing services to minority adolescents:

*… the question that kept coming throughout the training was, okay, a young person comes and asks a condom - “But what if I know that they’re gay?” [laughter] and it’s like, well, just give them the condom and, you know, they did not come to ask you “Should I be gay or not?”, they’ve come to ask you for a condom. Deal with the issue at hand. And, you know, let them go. And, “Oh, so, what should I do if I find out someone’s gay?” and I’m like “Just give the service that they’ve come for and let them go on their way.” But I think there’s that issue of interpretation of what does the law say, and what does it mean for services provision, and then you add on to that my own personal value system and what I think about those issues.* (Rutendo, from an (A)SRH organisation in Zimbabwe)

Others, however, spoke about their own lack of knowledge on issues of sexual orientation and gender identity, calling homosexuality ‘a new thing’ and cited this as a reason for not bringing these issues up in their work with adolescents:

*Even the NGOs that are operating in Malawi, not many of us know more about this issue.* (Patience, from an (A)SRH organisation in Malawi)

Many participants like Patience had difficulty naming “this issue”, suggesting an underlying discomfort. Rather than not knowing much about “this issue”, participants may hold problematic or negative views about sexual and gender minority adolescents.

Other participants were more forthright in expressing their disapproval, arguing that same-sex activity was immoral, or that sexual and gender minority adolescents ‘did not exist’ in their country, or area. Caroline is emphatic about her perception of Zimbabwe as a heterosexual nation:

*Discussing about lesbianism and preaching about it, you know, we can’t do that here. We don’t want to see all those young men and women that are into homosexuality. It’s not a thing that we can discuss here in Zimbabwe. Here in Zimbabwe we’ve got a stance, we don’t want it, people don’t want it.* (Caroline, from an (A)SRH organisation in Zimbabwe)

Similarly, Patrick, in Malawi sees same-sex activity as a product of the moral corruption of urban areas, which does not take place in modest rural areas:

*These things are happening, more specially in towns. People, the very same sex, they are doing the sex. Women sleeping with women. Men sleeping with men. It happens all in town, not in the community areas, [which are] very modest areas, no.* (Patrick, from an (A)SRH organisation in Malawi)

It was clear that the opinions of the participants reflect broader attitudes towards sexual orientation and gender identity in these countries. Many of the participants who were accepting of sexual and gender minority adolescents, both within LGBT organisations and in SRH organisations, spoke about how their environment was heavily infused with attitudes that discriminated against, and socially excluded adolescents based on their sexual orientation or gender identity:

*There is nothing in the law that criminalises homosexuality in Mozambique or even same-sex relationships in Mozambique, legally there is nothing hampering it, but socially there is an immense barrier.* (Victorino, from an (A)SRH organisation in Mozambique)

*…what I’ve also found in this country is that it’s the whole political rhetoric that then influences what happens on the ground. So, even our police find it easy to go and harass people and so forth because they know that, politically, no one is going to come down on them. Although, really, it’s not legal. Yeah.* (Rutendo, from an (A)SRH organisation in Zimbabwe)

In the professional experience of participants, such discrimination included bullying and violence at school and in other social spaces, the loss of friends and social networks, rejection by families and ejection from the family home, and experiencing discrimination when accessing public services, including healthcare.

### (A)SRH services in public health facilities

While public health facilities technically provide ASRH services, these services are typically not accommodating for sexual and gender minority adolescents. Participants from LGBT organisations in all five countries described how heteronormative public health facilities were:

*[LGBT adolescents] cannot even get the, even when we talk of the youth friendly facilities, even in those, they are not, they cannot, the people there, the training of the youth friendly facilities service providers does not include talking about sexual diversity, does not include the LGBTI people. It doesn’t. And so, it falls through.* (Munya, SRH consultant in Zimbabwe)

*I don’t want to have a broad brush that […] healthcare [in Zambia] is homophobic – that’s not the case – but how would youth know how to find that one practitioner that may, in fact, be sensitive and open to serving them? They wouldn’t necessarily know that. And, so, it’s very difficult but you could get shamed, shunned, reported; there’s nothing to protect you.* (George, a service provider in Zambia)

Consistently, participants attributed the deficiencies in service provision and the heteronormativity in public health facilities to health professionals’ training, or lack thereof when it comes to sexual and gender minorities, and adolescents in particular:

*We have nurses who complete the nursing program, but they do not have knowledge about issues related to gender identity and sexual orientation. Then appears someone in your office whose identity card has the name Antonio, however you see a transgender woman who is dressed differently from what the nurse expected for an Antonio to be dressed… We have cases of patients that have already been told that, ‘I am here waiting for Antonio and not Mary.’ The nurse just knows that Antonio has to dress as Antonio […] So here starts the first hurdle, instead of the nurse offering to start health services, they judge that individual because of the way they dress, this is where the problems start.* (Joao, from an LGBT organisation in Mozambique)

*And, then, we don’t really have, most of our medical personnel have not had the opportunity to be trained out of Zambia where you have the opportunity to interact with different kinds of patients. So, they don’t really know how to treat a trans person when they come in for a service, for example*. (Thandi, from an (A)SRH organisation in Zambia)

Furthermore, commodities for sexual and gender minority adolescents such as dental dams and lubricants were generally not available in public health facilities. Where private care exits, these may be available in a private pharmacy or a private clinic*.* Participants from LGBT organisations highlighted that the unavailability of commodities in public sector care was linked to the criminalisation of same-sex activity, and fears of ‘promoting homosexuality’:

*Dental dams and finger cots are kind of illegal […] because of its association with the LGBTI community. […] So if you are providing dental dams, it’s more like you're spearheading the act of homosexuality. So they are not usually accessible, you can’t find them, because that would be seen as […] trying to promote homosexuality. So you’ll never find it anywhere.* (David, from an LGBT organisation in Zambia)

The need for parental consent to access SRH services and commodities in public health facilities is a barrier for all adolescents wanting to access these services. Participants from LGBT organisations highlighted how this can pose particular challenges for sexual and gender minority adolescents:

*People have lost their family ties because they are homosexuals. And most of them are the youngsters that are not yet independent, who cannot stay on their own, who cannot pay for their own school fees, and some have even dropped out of school – we have so many people that have dropped out of school because their parents knew that they are homosexual and they say, ‘no, we don’t want a child like you.’ So, it’s a double dilemma that they can’t go to health services so they can’t access, let’s say condoms for safer sex. They can’t access condoms for safer sex at the health services, because they are underage and they can’t go with parental permission.* (Kennedy, from an LGBT organisation in Malawi)

As Kennedy explains, sexual and gender minority adolescents may be more likely to lose family support due to their disapproval of the adolescent’s sexual orientation or gender identity. As a result, they may not access education and health services that require parental consent.

### Heteronormativity in adolescent sexual and reproductive health services

Previous research has shown that confidentiality, youth-friendliness, a broad range of services, and affordable care are important factors in adolescents’ preferences to access sexual and reproductive health services and information from private service providers, NGOs, or the public sector [29]. It is unsurprising that confidentiality and youth-friendliness are especially important for young people, as they are widely perceived as violating religious and moral norms by engaging in sexual activity [30]. Urban adolescents may have a stronger preference for private providers, which are perceived as more confidential, or NGO providers, which are perceived as youth-friendly [31]. This means that NGOs play a pivotal role in providing SRH services to adolescents, particularly in urban settings. This is arguably even more the case for sexual and gender minority adolescents, whose SRH concerns require practitioners to be direct and detailed in the information that they provide, as illustrated by Tafadzwa’s description of the questions that practitioners should expect from sexual and gender minority youth:

*What are the risks that are involved when it comes to SRH if you decide to engage in sexual activity? What does the law of Zimbabwe say if you have anal sex? What is sodomy? If you are caught, what does it mean? And then we also go, what are the risks that are involved in you having unprotected sex? And what can you do to prevent if you decide to? Where do I get the condoms? Where do I get the lubricants?* (Tafadzwa, from an LGBT organisation in Zimbabwe)

This quote explains the level of detail and reassurance sexual and gender minority adolescents need when deciding where to access SRH services. In our entire sample of NGOs, however, none provided services tailored to sexual and gender minority adolescents. Participants recognised that there are very few, if any, SRH education and services for sexual and gender minority teenagers:

*[W]e do not have that system or recreational spaces where young people can go and speak or meet other young people to speak to about these things. So, it’s really a struggle, there's not space for LGBT young people to get together, there’s no environment created.* (Hilma, from an LGBT organisation in Namibia)

The reason for this gap in service provision among mainstream (A)SRH organisations is clear: pervasive heteronormativity in ASRH policy and provision. When we asked why they do not include sexual and gender minority teenagers in their service provision, Kelvin, from an SRH organisation in Zimbabwe, said: ‘*We’re just taking them as a homogenous group, like they’re all heterosexual*’. That this is an erroneous assumption was clear to those working in the LGBT sector, as highlighted by Kennedy, a representative from an LGBT organisation in Malawi:

*If you have the youth, the heterosexual youth that engage in the sex before the age of 18, why should we assume that we don’t have adolescents who are homosexual who are also engaged in sex before the age of 18?*

Heteronormativity creates a blind spot for sexual and gender minority adolescents, creating barriers to their ASRH access.

#### Sexual and gender minority stigma and criminalisation

Participants from (A)SRH organisations cited two major reasons upholding this heteronormative approach to ASRH services: the social stigma attached to homosexuality, and the criminalisation of same-sex activity. Stanley, who works at an (A)SRH organisation in Malawi, describes how these two issues converge to determine the content of SRH education and information materials for adolescents:

*[The penal code] has impacted that people don’t want to talk about these things and, also, we don’t have sufficient communication, IEC [information, education, communication] material to use in terms of disseminating this. Of course, physically, these IEC materials are produced by the government and, you know, proofread by the National AIDS Commission, so we don’t really have much of this information. So, they have an impact because no one wants to talk about it and we don’t have sufficient IEC materials in terms of doing, you know, anal safer sex.*

For Stanley, criminalisation in Malawi generates and compounds social stigma, and also means that the actual resources produced are subject to censorship – as they are produced by the state.

This was not the case in Malawi only. A number of participants across the five countries spoke about the challenges of including information about sexual orientation and gender identity into information materials. In Zambia, the control that government has in determining the content of SRH materials, including comprehensive sexuality education curricula, was evident:

*We were directed to […] remove those aspects that have got to do with lesbians and gays and so on from the peer education manual. So, we haven’t completed it yet, it’s still in the process of development, but we are warned to be […] cautious in terms of how we include it in the manual because the government will not sign it off if it finds that there are these issues that are included there.* (Cynthia, from an (A)SRH organisation in Zambia)

Beyond criminalisation (in four countries) and social stigma, discriminatory and judgmental attitudes of service providers in adolescent SRH organisations, as already described above, emerged as a third reason for not providing services to sexual and gender minority adolescents.

#### “Recruitment” panic: A barrier to LGBT organisations’ service provision

As a further complication, participants from LGBT organisations in countries where same sex activity was criminalised (Malawi, Namibia, Zambia and Zimbabwe) very firmly stated that they were governed by the legal age of majority (18 in all focus countries) in determining who they can serve. This is different from the age at which adolescents can legally access SRH services: age 16 in Zambia and Zimbabwe, but not specifically defined in Malawi and Namibia [6].

In contrast to (A)SRH organisations, LGBT organisations did not provide services because they were afraid that work with adolescents would lead to increased public scrutiny and opposition, and moral backlash to their overall work:

*As an organisation, we see the great need to do work on [ASRH]. […] But still, as a director, I feel completely uncomfortable when I see a child in school uniform in the office. […] The risk is quite high. [W]e would be attacked [for] the issue of ‘recruiting and promotion’. [… A]lso the fact that it can taint our mandate as an organisation and […] that religious groups shall attack and bring it down as a movement.* (Hilma, from an LGBT advocacy organisation in Namibia)

*It’s like a no-go zone […] during our meetings that we have with MSM clients, if we find that there is somebody who is below the age of 18, we ask them to […] leave.* (Blessing, from an LGBT advocacy organisation in Malawi)

‘Recruitment and promotion of homosexuality’ is not a criminal offence in any of the focus countries. Despite this, many participants were afraid of being accused of *“*recruit[ing] the youngsters into homosexuality” (Blessing, Malawi), of “initiating something” (David, Zambia), and thus of being “put in trouble” (Tafadzwa, Zimbabwe). While none of the relevant laws on same-sex activity actually outlaw work with adolescents, it was clear that LGBT organisations elected to only work with adults to avoid accusations that could damage their reputation or jeopardise their overall programmatic work.

Where organisations did provide education or services to teenagers, the interpretation of ‘recruitment and promotion’ also meant that they had to be careful in how SRHR education messages were framed:

*It’s just the language. We all know for a fact, when you write language like you're ‘promoting the advancement and well-being of LGBT people’. But we now know, we don’t use the term ‘promotion’, we just ‘advance the well-being’. So, it’s all about playing with language.* (Hilma, from an LGBT organisation in Namibia)

Hence, the universally accepted terminology of ‘health promotion’ is eschewed in order to avoid any potential association with notions of ‘promoting homosexuality’.

Mozambique, the only country in this research where same-sex activity is not criminalised, was the only country where the LGBT organisation representative did not mention the age of legal majority as defining access to services. However, even in this decriminalised context, sexual and gender minority youth are still highly stigmatised. Joao, a participant from a LGBT organisation in Mozambique, shared an experience that led them to determine that they will not work with teens younger than 15 years old. Despite the relative legal freedom, Joao describes how fear of being seen to recruit young people to homosexuality shapes LGBT organisations’ work:

*[A boy] posted a photo on Facebook in one of our programs. It was a social program, he took a picture and he had our t-shirt and posted the photo on Facebook and someone from his family saw the photo and [said] that [organisation’s name] was pushing it, and was promoting homosexuality to their child. As if their son’s sexual orientation was the product of him participating in our programmes. We have had several situations like these, it is why strategically, the organisation decided that we will reduce our involvement with younger beneficiaries, taking into account that individuals older than 15 years are more aware of what they are and what they do.*

This instance clearly describes how stigma can continue to impact sexual and gender minority adolescents’ access to services, even in a decriminalised setting.

Resultantly, educational material tailored to sexual and gender minority adolescents on ASRH, including sexuality, did not exist in any of the countries in this study. This means that sexual and gender minority adolescents in these countries have hardly any access to information about safer sex, or to resources that could help them develop a healthy relationship to their sexuality:

*[Sex between two men is] mentioned as something that happens, but […] there is no special mention of specific HIV prevention strategies for that group like, you know, use of lubricants or anything like that, no. It’s just mentioned that this can be there and, in the school material, it’s then mentioned that […] it is criminalised*. (Rutendo, from an (A)SRH organisation in Zimbabwe)

Also as a result of being unable to provide services to teenagers themselves, LGBT organisations started organising referral networks to adolescent SRH organisations. Often, this involved careful vetting of potential referral organisations, and required substantial education on issues of sexual orientation and gender identity for the service providers of these organisations before referrals could take place. Hilma and Tafadzwa described how such referral systems worked in Namibia and Zimbabwe, respectively:

*We are only since last year getting into the SRHR agenda because they excluded us for so long. [Adolescent SRH organisation] is the one that is working closely with us in the regions because they are the main service providers for young people, from 12 till 18 or above. Because at [adolescent SRH organisation], you can go in there and get your young child serviced with confidentiality guaranteed. [Adolescent SRH organisation] is also one of the friendliest partners that we have, that we always pick up the phone and say ‘we are dropping ten people now’. The director will leave whatever he is [doing and] attend to our needs. So, [adolescent SRH organisation] is the only enabling clinic in this country for young [LGBT] people.* (Hilma, from an LGBT organisation in Namibia)

*What we do is, […] if there are people from the LGBTI community that are under 18, we do have partners that we work with where we are able to refer them for specific services. If it’s, for instance, they want counselling because a lot of young adults, they struggle with understanding their sexuality, we make sure that we do it with the [counselling service…] If they feel that they need somebody from the [LGBTI] community to provide the counselling, we do have people that have been trained to also have skills in family therapy […] So sometimes we go in and actually do the counselling there because it’s an established entity and also for safety reasons.* (Tafadzwa, from an LGBT organisation in Zimbabwe)

These referrals appear to work for adolescents because (A)SRH organisations are perceived differently to LGBT organisations. Importantly, (A)SRH organisations are able to frame SRH services to sexual and gender minority adolescents within a public health approach rather than an advocacy approach, and thus provide them services without the risk of being perceived to ‘promote and recruit,’ as illustrated here:

*Because of the fact that our emphasis is on health, we are very comfortable to defend that approach […]. And nobody can argue that […] we are an advocate for the LGBT sector. We accept [LGBT adolescents] from a health perspective and we provide the service to the young person, to the LGBTI person on the basis of HIV prevention. We’d rather help this person, empower this person to help him or herself and therefore in the process reduce [HIV risk].”* (Charles, from an (A)SRH organisation in Namibia)

However, this referral strategy is not sufficient to ensure access to SRH services for all sexual and gender minority adolescents. The referral networks between LGBT and (A)SRH organisations that our participants spoke about were based on individual relationships and networks. Without agreed-upon institutional commitments, access to SRH services for sexual and gender minority adolescents is dependent not only on the brokerage of the LGBT organisation, but also on individual providers within the (A)SRH organisation. While this approach might work well when the individuals are sympathetic, it raises questions about the sustainability and longevity when (A)SRH organisations experience personnel changes – especially given the existing discriminatory attitudes of some adolescent organisations’ staff.

### The impact of criminalising laws on access to services

Participants spoke at length about how the current criminalisation of same-sex activity in four of the study countries impacts access to SRH services for sexual and gender minority adolescents. While none of the laws pose a direct legal barrier to services, the legal framework contributed to an environment of state-endorsed homophobia, in which public healthcare providers, and at times even private or NGO service providers, could refuse to provide services to sexual and gender minority adolescents. There were five distinct ways in which criminalising laws contributed to the barriers to sexual and reproductive health services that sexual and gender minority adolescents face.

First, Kennedy from Malawi (LGBT organisation) recounted that healthcare providers had reported sexual and gender minority patients who had sought SRH services to the police, because healthcare providers were under the (erroneous) impression that they had a duty to report:

*We’ve had a report where some medical providers have reported the patient to the police. You see, you get to the confidential room, you tell them your problem and you open up because you expect the doctor to be confidential about issues, so you open up with them to say ‘ok, I’m gay, and I have sex with several guys, and I have this problem,’ and then there was a situation where the medical person said ‘ok, fine, just wait for me here’ and then went out, made a phone call to the police: ‘I have a homosexual here.’ Something like that. It happened in Malawi. And you can imagine that such a thing went all over the MSM networks, telling themselves, ‘Guys, don’t you ever go to such-such a hospital; they are going to arrest you; this is what happened to me.’*

Second, as Kennedy’s quote highlights, the fear of potentially being reported prevents sexual and gender minority adolescents from seeking services. This reluctance compounds with other reasons for which adolescents might not want to seek services, such as fear of age-related judgmental attitudes from healthcare and other service providers. Kennedy elaborated:

*Despite the fact that […] Malawi’s trying […] to promote the youth friendly services, the laws [criminalising same-sex activity] have been there before and they are still at the back of so many of our minds, including the service providers. So, you find out that even when the youth go there and go to places which are termed to be ‘youth friendly service providers’, they still find some other service providers who […] say ‘How could you do that as young as you?’ So, at the same time, even if we, the young MSM, were to access the services at the youth friendly service centres, they will also experience something similar to that, to say, ‘How do you manage to be having sex through your anus?’*

Third, criminalising laws contribute to and validate social stigma related to non-heteronormative sexual orientation and gender identity. Whether or not these laws are enforced, the fact that they exist is often used to justify discriminatory, harmful and exclusionary practices against sexual and gender minority adolescents. Shelton, who works for an LGBT organisation, explains how this played out prior to decriminalisation in Mozambique:

*I would just say that there has never been a habit […] of arresting people because they have sex with a same-sex partner. The point is that there was a law [that criminalised same-sex activity], if someone wanted to they could use the law, but in reality it was not used, it was not a real prison or a real penalty because of this, however Mozambique has a context with stigma for those who have sex with someone of the same sex.*

Fourth, misinterpretation of criminalising laws perpetuates false perceptions that justify discriminatory practices. For example, some participants from (A)SRH organisations were under the impression that they could not talk about sexual orientation or gender identity during comprehensive sex education because they would be ‘promoting’ criminalised behaviour. Evelyn, who works at an (A)SRH organisation in Zimbabwe, was even under the impression that some HIV prevention commodities (dental dams and lubricants) were also criminalised:

*You don’t have commodities for young people who have got a different sexual orientation in Zimbabwe; you cannot come through our borders with that kind of material.*

This is actually not true – Tafadzwa, from a Zimbabwean LGBT organisation, told us that the organisation had received a shipment of condoms and lubricant, paid for by the Ministry of Health under a Global Fund-funded ‘key population’ programme. Thus, Evelyn’s erroneous perception illustrates how social stigma and criminalising laws collide and lead to misinformation. This is also illustrated in an anecdote that Kennedy from Malawi (LGBT organization) shared about healthcare providers refusing to provide services to sexual and gender minority adolescents:

*Sometimes even the service providers themselves feel like, ‘Ok, if I’m going to provide the services to a homosexual, it means I’m condoning homosexuality and therefore I may be arrested as well,’ for helping someone to be a homosexual or to stay a homosexual, something like that.*

If and when such cases of discrimination come to the attention of an LGBT organisation, the organisation, acting on behalf of their client, often directly challenges the individual healthcare provider’s refusal to provide services. However, in cases where the patient is an adolescent, organisations are limited in their response due to the concerns around perceptions of ‘recruitment’ and ‘promotion of homosexuality’ discussed in earlier sections.

Fifth, criminalisation contributes to confusion and misconceptions regarding healthcare professionals’ duties given the contradictions between laws which criminalise same-sex behaviours and policies which promote HIV service provision to ‘key populations,’ including sexual and gender minorities. Evelyn from Zimbabwe said that this “discrepancy” means that, against what the law says, “we are giving [LGBT people] whatever they want, we are giving them access”. Thandi, from a Zambian (A)SRH organisation, summarised her perception of the conflicting obligations of Zambian healthcare providers, echoing once more the need for trained, sensitive providers:

*We should be having focused programming on health for this group but, like always, there was the push back to say, ‘there’s this law that does not allow.’ So, if you’re creating health programs for this group, you are in essence going against what the law provides for, because that then becomes an illegal service. But, also, the fact that who will be able to provide those services, because you need someone that understands but also that is non-judgemental to be able to provide the services.*

Kennedy, from an LGBT organisation, observed the same impediment in Malawi:

*[…] even the health service provider, themselves, […] the legal framework has affected what is contained in their training […] So you find out that they come back from the training institutions without knowing how they are going to attend to the needs of the ‘vilest’ people, including MSM [men who have sex with men] or including LGBTI.*

While this conflict between law and policy can be problematic, it can also serve as an opportunity to provide sexual and gender minority adolescents with SRH care:

*I think if we were to smoothen things on the legal side, I think it would be easier for the service provider on the health side to then work with the adolescent. Sometimes, you want to engage them which is something that I think is something that really should be done, so you want to engage them and put in place certain intervention because of the legal issue behind the scenes, you may engage them but you may not be able to provide their service. So, yeah, that’s a serious challenge.* (Elvis, Zimbabwean policy maker)

According to this policymaker, public health framing is a potential loophole to ensure SRH service provision to sexual and gender minority people, further arguing that law reform should be considered in order to harmonise law and public health policy.

## Discussion

In this paper, we presented data from in-depth interviews with 51 participants working in (A)SRH and LGBT organisations from five countries in Southern Africa. Our findings highlight the double-marginalisation of sexual and gender minority adolescents: they are excluded from LGBT-specific services aimed at adults, and at the same time, they are excluded from heteronormative (A)SRH services. This double-marginalisation is in large part due to, and reinforced by, laws that criminalise consensual sexual behaviour between partners of the same sex/gender, regardless of their age. However, the findings also reveal the role that homophobia and stigma play in these processes, including in Mozambique where same-sex/gender sexual behaviours have been decriminalised.

Our findings are subject to a number of limitations. First, we did not speak to sexual and gender minority adolescents themselves, to avoid placing them in a more vulnerable position. It is important to bear in mind that our findings are based on the perspective of service providers. However, we believe that service providers might actually have been better able to provide insight into pathways and policy-related barriers, as their work allows them to compare and collect individuals’ experiences. Second, we focused our work on urban areas, as this was where most NGOs were located. Our findings might therefore neglect the specific barriers related to geographical location that sexual and gender adolescents in rural areas face. Based on the literature, it can be assumed that attitudes around sexuality might be even more conservative [30], and access to ASRH services even more sporadic [32] in areas far from urban centres. Third, we did not speak to healthcare providers, which limits our insight into healthcare providers’ attitudes and knowledge on sexual and gender minority adolescents. The impression that our participants gave of conservative and judgmental healthcare provider attitudes is confirmed by literature [14, 33], but further research will need to explore the relationship between provider attitude, knowledge, and socio-legal context.

Despite these limitations, our findings resonate with the literature. Other authors have recently pointed to the erroneous assumption of heteronormative homogeneity of adolescents in SRH policy: Judhistari and colleagues [34] critique the narrow view that such policies take, and Hindin and Fatusi [35] show how narrow ideas of who adolescents are fail to recognise the diverse needs within adolescent populations (due to, among others, dis/ability, sexuality and socio-economic status). Our findings highlight the consequences of heteronormative policies, which result in a complete lack of sexual and reproductive healthcare services for sexual and gender minority adolescents.

There are compelling arguments that show why sexual and gender minority adolescents in particular need access to such services: research from South Africa shows that HIV prevalence is higher among adult men who have sex with men than in the general population [36], and that 10% of adult women who have sex with women self-report that they are living with HIV [37]. Given that, in Southern Africa, 15-24 year olds currently have the highest HIV prevalence [3], this means that in all likelihood, sexual and gender minority adolescents carry a significant burden of HIV infection. Equally crucial is the mental health and well-being of sexual and gender minority adolescents, which is included in comprehensive definitions of sexual health [38]: international studies show that sexual and gender minority adolescents tend to have higher rates of depression and suicidal ideation and attempts, often because of their experiences of social exclusion and marginalisation due to their sexual orientation and gender identity [39]. A South African study evidences that sexual minority youth have higher levels of partner-perpetrated violence, and higher levels of depression, traumatic stress and substance use when compared to heterosexual matched peers [28]. Research further shows that structural stigma, including through criminalising laws, leads to worse mental health outcomes among sexual minority groups [26]. In brief, this means that sexual and gender minority adolescents in countries with criminalising laws might be in dire need of the services that are denied to them.

When the law criminalises consensual same-sex/gender sexual activity, our findings have shown that conversations with sexual and gender minority adolescents seeking SRH services, if and when they happen, tend to focus on the legal provisions and potential punishments. This has important implications for how sexual and gender minority adolescents can, or cannot, conceptualise consensual sex. If all sex they have is illegal and no SRH educational material targeting sexual and gender minority adolescents exists, these adolescents are not provided with vital information about sexual violence within same sex/gender sexual encounters and relationships. Research points to the importance of nuanced, detailed conversations about the nature and context of consent with adolescents in order to develop healthy ideas about sex and sexuality, and to prevent sexual and other forms of violence [40]. For sexual and gender minority adolescents, then, the illegality of any sex that they may engage in complicates these conversations and runs the risk of shifting the focus of the conversation on the legal parameters of sexuality rather than on nuanced discussions of mutual consent.

Further, due to the fluidity of punitive and stigmatising discourses such as those about ‘recruitment and promotion of homosexuality’, in countries where same-sex behaviours are criminalised, and even those where they are not, working with adolescents takes on an added dimension of risk for LGBT organisations. While not a law in the five study countries, the offence of ‘recruitment’ does exist in other African countries (for example, Uganda [41, 42]). This speaks to both the ability of homophobic legal discourses to travel on the continent and embed itself within and perpetuate societal homophobia, and the way in which they may override enabling local legal frameworks in the provision of ASRH services. Thus, LGBT organisations are wary of accusations of recruitment of minors due to societal homophobia and not because of, but despite, the legal framework.

Over the past 10 years, advocacy for better healthcare services and against the criminalisation of LGBT populations have been more successful when they were framed as public health arguments [43]. In other words, governments were more likely to concede some rights to sexual and gender minority people if these rights were linked to health concerns. For example, men who have sex with men and transgender individuals are now recognised as ‘key populations’ in many African countries, even if same-sex activity remains outlawed. In these countries, MSM and transgender people are explicitly mentioned in national policy documents by Ministries of Health, usually related to HIV prevention, and, with financial support from international funding sources such as the Global Fund, these Ministries of Health provide services for ‘key populations’. As Epprecht [43] shows, the careful, nuanced argument that was successful in these cases focused on public health rather than ‘LGBT rights’, and thus allowed governments to provide services to ‘key populations’ without requiring a public declaration that they support rights for sexual and gender minority people. Our findings caution, however, that these ‘key populations’ are usually conceptualised only as adults, and that sexual and gender minority adolescents are thus not included in programming and service provision. Our findings underscore the need to acknowledge adolescents as part of key populations [44], with special emphasis on young people’s heterogeneity and particular vulnerabilities that Baggaley et al. [45] recently articulated.

Policy development, however, will need to be accompanied by mandatory education and training for healthcare and other service providers on serving sexual and gender minority adolescents, in order to ensure that key population policies are actually implemented for adolescents. Our findings highlight the sexual orientation- and/or gender identity-related barriers that sexual and gender minority adolescents face when accessing SRH services. These barriers compound age-related barriers that are already reported in the literature: nurses’ judgment about sexuality at young ages [30], and discretionary service provision based on nurses’ perceptions of an adolescent patient’s ‘worth’ [7]. There is a clear need for policymakers and service providers to be aware of, and sensitive to, the double-marginalisation experienced by sexual and gender minority adolescents, based on both their age and sexual orientation and/or gender identity.

## Conclusion

The African Commission on Human and People’s Rights, in its resolution 275 [46] and a recent report [47], has emphasised the need to protect people against violence and other human rights violations on the basis of their real or imputed sexual orientation or gender identity [46, 47]. The report included a call for states to revise legislative frameworks that criminalise consensual same-sex/gender sexual behaviour. Our findings provide further evidence for the need for legislative reform by highlighting the deleterious consequences of denying SRH service provision to sexual and gender minority adolescents, particularly in countries with criminalising frameworks. The combination of laws that criminalise same-sex/gender sexual activity and societal homophobia effectively exclude sexual and gender minority adolescents from existing (A)SRH services, leaving this vulnerable group without access to information about their sexuality, about sexual health, and about how to protect themselves from HIV.

## Abbreviations

(A)SRH organisation: Organisations either providing specific adolescent sexual and reproductive health (ASRH) services, or general sexual and reproductive health services that are also open to adolescents
AIDS: Acquired immune deficiency syndrome
ART: Antiretroviral therapy
ASRH: Adolescent sexual and reproductive health services
HIV: Human Immunodeficiency Virus
IEC: Information, education, communication
LGBT(I): Lesbian, gay, bisexual and transgender (and intersex)
MSM: Men who have sex with men
NGO: Non-governmental organisation
SADC: Southern African Development Community
SRH(R): Sexual and reproductive health (and rights)

## Footnotes

1 In an attempt to be precise about what the existing laws criminalise, activists and scholars specify that it is ‘same-sex activity’ that is contentious, rather than gay/ lesbian/ bisexual or transgender identity. We agree, and at the same time are aware that the term ‘sex’ (denoting physical attributes) is inappropriate to refer to gender diversity and gender non-conformity, and therefore also include the term ‘gender’ (denoting people’s identity, rather than their bodily characteristics).

2 While LGBT (lesbian, gay, bisexual, transgender) holds a lot of recognition value in Southern Africa, we consciously decided to rather use the term ‘sexual and gender minority adolescents’. This is not to assume that the experiences of people who identify as lesbian, gay, bisexual or transgender are the same – in fact, we are mindful of the heterogeneity, and the various, intersecting ways in which people who identify as LGBT are marginalized. Rather, we use this descriptor to highlight the commonalities of the source of this marginalization: heteronormative norms that result in the marginalization of all people whose identities or practices defy these norms.

3 We decided to retain the terminology used by organisations themselves, and employed by our participants, and thus use the term ‘LGBT’ when referring to organisations, or when it is used in direct quotes.
